# COHORT PROFILE: The Complications of Long-Term Antiretroviral Therapy study in Uganda (CoLTART), a prospective clinical cohort

**DOI:** 10.1186/s12981-017-0154-y

**Published:** 2017-05-04

**Authors:** Billy Nsubuga Mayanja, Ivan Kasamba, Jonathan Levin, Ivan Namakoola, Patrick Kazooba, Jackson Were, Pontiano Kaleebu, Paula Munderi, on behalf of the CoLTART study team, Billy N. Mayanja, Judith Nalwadda, Gladys Nakibuuka, Harriet Namugenyi, Patrick Kazooba, Rosemary Lubega, Annet Mugisha, Apophia Tereka, Apuuli Kalyebara, Arthur Namara, Diana Nakitto, Deus Wangi, Fred Nume, George Ssemwanga, Gertrude Nabulime, Gladys Nassuna, Gloria Lubega, Ivan Namakoola, Joseph Lutaakome, Lillian Generous, Lydia Matama, Rosemary Massa, Salome Tino, William Nakahima, Anne A. Kapaata, Brian Magambo, Chris Parry, Frederick Lyagoba, Jamirah Nazziwa, Maria Nannyonjo, Edward Muhigirwa, Faith Wamalugu, Florence Kabajuma, Hope Grania Nakazibwe, Jackson Were, Joan Bwandinga, Juliet Bukenya, Member Zephyrian Kamushaaga, Peter Hughes, Peter Nkurunziza, Priscilla Agatha Balungi, Simon Mukasa, Sureyah Nassimbwa, Tobias Vudriko, William Senyonga, Willyfred Ochola, Annet Nakimbugwe, Catherine Nampewo, Doreen Nambuba, Erima Naphtali, Grace Barigye, Irene Nakamanya, Ivan Kasamba, Jonathan Levin, Joseph Kahwa, Joy Namutebi Matovu, Lillian Namayirira, Ruth Namulindwa Lubega, Sandra Nabalayo, Solomon Kaddu, Paula Munderi

**Affiliations:** 1 MRC/UVRI Uganda Research Unit on AIDS, P.O. Box 49, Entebbe, Uganda; 2 MRC Tropical Epidemiology Group, London School of Hygiene and Tropical Medicine, Keppel Street, London, WC1E 7HT United Kingdom; 3 Department of Clinical Research, London School of Hygiene and Tropical Medicine, Keppel Street, London, WC1E 7HT United Kingdom

**Keywords:** Cohort profile, Antiretroviral therapy, Metabolic abnormalities, Renal complications, HIV, Uganda

## Abstract

**Background:**

Antiretroviral therapy (ART) improves the survival and quality of life of HIV-positive individuals, but the effects of long-term ART use do eventually manifest. The Complications of Long-Term Antiretroviral Therapy cohort study in Uganda (CoLTART) was established to investigate the metabolic and renal complications of long-term ART use among Ugandan adults. We describe the CoLTART study set-up, aims, objectives, study methods, and also report some preliminary cross–sectional study enrolment metabolic and renal complications data analysis results.

**Methods:**

HIV-positive ART naïve and experienced adults (18 years and above) in Uganda were enrolled. Data on demographic, dietary, medical, social economic and behaviour was obtained; and biophysical measurements and a clinical examination were undertaken. We measured: fasting glucose and lipid profiles, renal and liver function tests, full blood counts, immunology, virology and HIV drug resistance testing. Plasma samples were stored for future studies.

**Results:**

Between July 2013 and October 2014, we enrolled 1095 individuals, of whom 964 (88.0%) were ART experienced (6 months or more), with a median of 9.4 years (IQR 7.0–9.9) on ART. Overall, 968 (88.4%) were aged 35 years and above, 711 (64.9%) were females, 608 (59.6%) were or had ever been on a Tenofovir ART regimen and 236 (23.1%) on a Protease Inhibitor (PI) regimen. There were no differences in renal dysfunction between patients on Tenofovir and Non-Tenofovir containing ART regimens. Patients on PI regimens had higher total cholesterol, lower high density lipoprotein, higher low density lipoprotein, higher triglycerides, and a high atherogenic index for plasma than the non-PI regimen, p = 0.001 or < 0.001. Patients on Non-PI regimens had higher mean diastolic hypertension than patients on PI regimens, p < 0.001.

**Conclusions:**

Our finding of no differences in renal dysfunction between patients on Tenofovir and those on Non-Tenofovir containing ART regimens means that Tenofovir based first line ART can safely be initiated even in settings without routine renal function monitoring. However, integration of cardiovascular risk assessment, preventive and curative measures against cardiovascular disease are required. The CoLTART cohort is a good platform to investigate the complications of long-term ART use in Uganda.

## Background

Since the Universal roll-out of antiretroviral therapy (ART) in 2004, access to and availability of ART has improved. By June 2016, globally 18.2 million HIV-positive people were receiving ART, up from 15.8 million in June 2015 and 7.5 million in 2010 [1]. In Uganda, 750,896 (50%) HIV-positive people were receiving ART by December 2014 [2]. Although in industrialised countries ART has been used for over 20 years, in sub-Sahara, ART availability in public health facilities only began around 2004. In Uganda, reports suggest that the prevalence of non-infectious diseases like diabetes mellitus, and cardiovascular morbidity and mortality are increasing [3, 4].

Long-term ART use is associated with metabolic, cardiovascular, hepatic, renal, bone, bone marrow and other complications or toxicities [5–9]. These complications of ART are variably associated with all major classes of ARVs. The three most common metabolic abnormalities that are related to ART are dyslipidaemia, lipodystrophy and dysregulation of glucose metabolism [5–9]. Renal impairment among HIV-positive individuals may be due to HIV associated nephropathy, co-infections and co-morbidities, or renal toxicity from ART and concurrent medications [10, 11]. Due to its cost, in resource limited settings Tenofovir had been reserved for use with Protease inhibitors in second line ART regimens. The WHO recommendation to use Tenofovir in first line ART regimens has been widely adopted by ART programmes in Africa including Uganda, Kenya and Tanzania [12–15]. However, Tenofovir is often associated with renal toxicity, manifesting as a decline in estimated glomerular filtration rate (eGFR), proximal renal tubular dysfunction and acute renal failure especially among patients with risk factors for kidney disease [16–23]. It has been suggested, that patients of African origin are at higher risk for HIV associated kidney disease [24], a suggestion that remains to be clarified by studies conducted in Africa. Therefore, for long-term HIV care in Africa, health workers need research-based evidence on renal function beyond 5 years of Tenofovir containing ART, and how these patients should be monitored.

In sub-Saharan Africa, there is scarce literature on the long-term impact of ARVs toxicities among African populations. Current ART guidelines in sub-Saharan Africa are therefore based on evidence from industrialised country settings. However, there are differences between industrialised countries and sub-Saharan Africa in terms of patient profiles, background risk factors for co-infections, co-morbidities, ART regimen choices and drug toxicities. In 2013, the Complications of Long-Term Antiretroviral Therapy (CoLTART) cohort was therefore established by the MRC/UVRI Uganda Research Unit on AIDS (MRC/UVRI Uganda Unit) as a platform for studying the complications of long-term ART among Ugandan HIV-positive adults. The study aimed at examining the metabolic and renal complications of long-term ART use among Ugandan HIV-positive adults aged 18 years and above. In this paper, we describe the CoLTART study set-up, aims, objectives, study methods, and also report some preliminary cross-sectional study enrolment renal and metabolic complications data analysis results.

## Methods

### The CoLTART study design and settings

The CoLTART study was a prospective clinical cohort conducted at two study clinics; (a) the former Development of Antiretroviral therapy in Africa (DART) Trial study clinic in Entebbe [25], and (b) the former Rural Clinical Cohort (RCC) study clinic in Kyamulibwa, approximately 120 kilometres from Entebbe [26]. The MRC/UVRI Uganda Unit’s Head offices and central Clinical Diagnostic Laboratory Services (CDLS) are located in Entebbe.

### CoLTART study aims and objectives

The CoLTART study aims were to study the metabolic and renal complications of long-term ART, and the long-term treatment outcomes of a triple nucleoside first ART regimen. The primary objectives were to compare the: (a) mean values for cardio-metabolic markers among patients on a Protease Inhibitor (PI)-containing ART regimen and those on a non PI-containing regimen. (b) Mean values of proximal renal tubular function and eGFR among patients on a Tenofovir (TDF) containing regimen and those on a non-TDF containing regimen.

#### CoLTART study participants

**Fig. 1:**
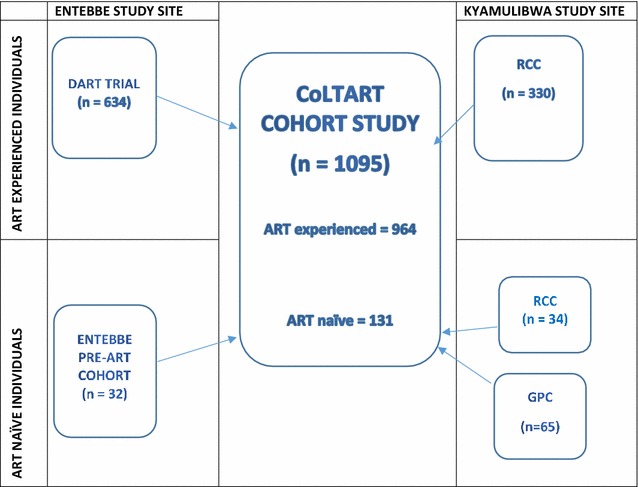
Sources of enrolled CoLTART cohort study participants

Between July 2013 and October 2014, CoLTART study participants (Fig. 1) who were HIV-positive adults (aged 18 and above) on ART were recruited from; (a) the former DART Trial Cohort, which was a randomised clinical trial of monitoring strategy for the management of ART in adults with HIV infection in Africa established in 2003 [25]. (b) The former RCC which was an open clinical cohort established in 1990 to study the natural history of HIV infection [26] and later the impact of ART after its introduction in 2004 [27–30]. ART naïve HIV-positive study participants, but who were eligible to initiate ART were enrolled from: (a) the RCC, (b) the General Population Cohort that was established in 1989 in rural southwest Uganda to examine the dynamics of the HIV epidemic [31] and (c) the Entebbe Pre-ART cohort that was established in 1995 as a double blind, randomised and placebo controlled trial of a 23-valent pneumococcal polysaccharide vaccine in HIV-positive adult Ugandans [32]. ART was provided according to existing National ART guidelines [13, 33]. The study included HIV-positive adults (aged 18 years and above), who were on ART or eligible to initiate ART, not participating in another study, who were willing to undergo regular clinical, biophysical and laboratory evaluation and to complete a study questionnaire. Individuals who were too sick to undergo study procedures, unable or unwilling to give informed consent were excluded from the study.

#### Participants’ enrolment and follow-up

Consenting eligible individuals were enrolled, and requested to fast from midnight of the day prior enrolment, and they received a drink and snack after the blood draw and biophysical measurements. We measured the body weight using the Seca digital measuring scale, height using a portable Seca 213 Leicester stadiometer and body circumferences using a non-stretchable Seca 201 Ergonomic Circumference Measuring tape (Table 1). Details of the methods for taking biophysical measurements and calibrating the equipment used have been described elsewhere [34]. Blood pressure and pulse rate were measured using the Omron M6 comfort automatic blood pressure monitor. At enrolment and six monthly follow-up visits, a modified WHO STEPS cardiovascular risk data surveillance questionnaire was administered to collect the relevant data [35]. Every three months, participants renewed their prescriptions for ART and/or cotrimoxazole or Dapsone prophylaxis and adherence data was collected using self-report and pill count. Every 6 months (Table 1), participants attended for scheduled follow-up visits, and they fasted as done at enrolment, and the data collected were recorded on a follow-up questionnaire and clinical examination form. At these 6 monthly visits, participants also renewed their medicines prescriptions and adherence data was collected. Participants who fell sick in between the scheduled visits attended the study clinics to receive medical care.

**Table 1:** Measurements and data collected in the CoLTART cohort

Phase	Data collected at enrolment
Baseline July 2013	Demographic data and self-reported social economic status Medical, dietary and socio-economic and behavioural risk factors Biophysical measurements: weight, height, blood pressure, pulse rate; waist, hip and mid-upper arm circumferences Clinical examination Antiretroviral therapy and cotrimoxazole/dapsone adherence *Laboratory measurements data* *Biochemistry* (i) Fasting blood lipid profiles: total cholesterol, triglycerides, low density lipoproteins, high density lipoproteins (ii) Fasting blood glucose (iii) Renal function tests: urine strip testing, phosphates and creatinine Serum phosphates, creatinine and urea (vi) Liver function tests: alanine transaminase, aspartate transaminase, alkaline phosphatase and bilirubin *Haematology and immunology* (i) Full blood counts (ii) CD3, CD4 and CD8 cell counts *Virology* (i) HIV viral loads (ii) HIV drug resistance testing—when indicated *Serology* (for future testing) Serum for Hepatitis B virus serology and plasma aliquots stored at −80 °C
6 monthly Follow-up January 2014	*Data collected at follow up* Biophysical measurements Medical, dietary and socio-economic and behavioural risk factors Clinical examination Antiretroviral therapy and cotrimoxazole/dapsone adherence *Laboratory measurements* Fasting blood glucose and lipid profiles Renal and liver function tests Full blood counts and immunology—CD3, CD4 and CD8 HIV viral loads HIV drug resistance testing—when indicated Whole blood for genomics studies stored at −80 °C (once)

### Laboratory procedures

#### Specimen collection and storage

At enrolment and follow-up visits, about 10–15 ml of venous blood were collected in three tubes as follows: (a) 4 ml of whole blood in an EDTA tube for haematology, immunology and viral loads measurements. (b) 5 ml plain serum tube blood to measure fasting serum lipids; renal and liver function tests. (c) 4 ml Sodium fluoride tube blood to measure fasting blood glucose. At enrolment only, serum for hepatitis B serology and two aliquots of plasma were stored at −80 °C for future testing. Blood tubes were labelled with a unique individual study and laboratory number and transferred under appropriate conditions to the CDLS laboratory for analysis. At one follow-up visit, a 4 ml of whole blood sample in an EDTA tube was stored at −80 °C for future genomics testing. Participants with viral loads of 1000 copies/ml or higher had antiretroviral drug resistance testing done. Participants (excluding women in their menses) provided a fresh midstream urine specimen in a plain sterile container, that was later portioned in two plastic centrifuge tubes (one plain for urine creatinine and the other acidified for urine phosphates measurements), and a dipstick strip test was done on the urine remaining in the container.

#### Laboratory testing

All samples were analysed in the MRC/UVRI Uganda Unit’s CDLS laboratories. The Clinical chemistry analyser, COBAS Integra 400 *plus* (Roche Diagnostics) was used to measure fasting serum lipids and blood glucose, and renal and liver function test parameters. Full blood counts were measured using the Coulter AcT5 Diff CP Analyser (Beckman Coulter, USA). CD4 cell counts were measured using either the FACSCount or FACSCalibur machine (Becton–Dickinson, USA). Plasma HIV-1 RNA was quantified using the COBAS Ampliprep/Taqman V2.0 HIV-1 viral load assay (Roche Molecular Diagnostics [RMD], NJ, USA) with a lower detection limit of 20 copies/ml. For urine strip testing, we used the Siemens Multistix 10SG and read with Clinitek Status Analyzer (Siemens Healthcare Diagnostics). For ARV drug resistance, PCR and sequencing reactions were conducted and the sequences submitted to the Stanford University HIV Drug Resistance database. The surveillance drug resistance mutations were identified using the 2009 WHO list for surveillance of transmitted drug resistances [36], using the Stanford calibrated population resistance analysis tool version 5.0 beta [37]. Sequences with genetic mixtures of wild-type and mutant sequences at amino acid sites that code for SDRMs were considered to be drug-resistant.

#### Laboratory data quality assurance

Standard operating procedures and internal quality measurements ensured internal quality control. The United Kingdom National External Quality Assurance Service, College of American Pathologists and the Royal College of Pathologists of Australasia were used for External quality Assurance for both haematological and biochemistry assays. Virology Quality Assurance Scheme (Rush University, Chicago, IL) was used for External Quality Assurance for virological assays.

### Data management and statistical methods

Data was collected on the case report forms which together with the Laboratory result forms were sorted, batched and submitted to the Statistics section for data entry. Data was managed in accordance with the ICH-GCP data management principles using a single Ms. Access study database using common identifiers to incorporate relevant retrospective data from the DART Trial Cohort and RCC databases. All the data was backed up on a central Unit server with a password restricted access. Enrolment statistical analyses were done in STATA 13 (Stata Corporation, College Station, USA). Participants’ socio-demographic and economic characteristics, lifestyle and anthropometric as well as clinical, history of diseases and ART exposure were examined across study sites and by sex. Renal function outcomes were determined using measured renal function tests, and calculated Fractional Tubular reabsorption of phosphates and estimated glomerular filtration rate (eGFR). Different formulae were used to calculate eGFR; (a) the Cockcroft-Gault formulae with and without body surface area adjustment, (b) the Modified Diet in Renal Disease formulae with race adjustment and (c) the Chronic Kidney Disease Epidemiology.

Abnormal renal function outcomes were defined as: serum urea of more than 11.9 mmol/l, serum creatinine of more than 109 μmol/l, Fractional Tubular phosphates reabsorption of less than 82% and eGFR less than 60 ml/min/1.73 m^2^, Diabetis mellitus as a measured fasting blood glucose more than or equal to 6.4 mmol/l or history of and or being on medications for diabetis mellitus. Hypertension was a measured systolic blood pressure (SBP) of more than or equal to 140 mmHg or diastolic blood pressure (DBP) more than or equal to 90 mmHg or any history of and or being on medications for hypertension. The renal function outcomes were compared across the Tenofovir exposure groups: (i) non-Tenofovir containing ART regimen (Non-TDF), (ii) Tenofovir containing ART regimen (TDF-ART) and (iii) previously on Tenofovir containing ART regimen but stopped (TDF-stopped). Mean values of renal function outcomes were compared by Tenofovir exposure using general linear models. General linear regression models adjusted for several factors were used to compare mean values of renal function outcomes across groups of Tenofovir exposure categories. The mean values of SBP, DBP, total serum cholesterol, high density lipoprotein, low density lipoprotein, triglyceride, atherogenic index of plasma and fasting blood glucose and Framingham risk score were compared between PI-based and non-PI based ART regimens using general linear regression models, adjusted for duration on ART and other factors.

## Results

Between July 2013 and October 2014, we assessed 1108 HIV-positive individuals for study eligibility, of whom 1095 (98.8%) were enrolled into the study. At the Entebbe site, 673 individuals were assessed for study eligibility and 666 (99.0%) were enrolled and 7 (1.0%) were not enrolled because; 3 (0.4%) were participating in another study, 3 (0.4%) did not return for enrolment while 1 (0.1%) died before enrolment. At the Kyamulibwa site, 436 individuals were assessed for study eligibility and 429 (98.4%) were enrolled, 7 (1.6%) were not enrolled because 3 (0.7%) requested for transfer to other ART care centres, 1 (0.2%) had mental illness, 1 (0.2%) declined consent for participation, 1 (0.2%) defaulted and 1 (0.2%) died before enrolment. Of the 1095 individuals enrolled, 964 (88.0%) were ART experienced (6 months or more) with a median of 9.4 years (IQR 7.0–9.9) on ART. Overall, 968 (88.4%) were aged 35 years and above, 711 (64.9%) were females, 608 (59.6%) were or had ever been on a Tenofovir containing ART regimen and 236 (23.1%) on a Protease Inhibitor containing ART regimen. Participants at the urban (Entebbe) site were older, mean age (SD); 46.1 (8.1) vs 42.7 (10.6) years and had a higher education level; 54.3% vs 15.4% had attained secondary level education and above. Whereas most of rural participant were peasants (69.5%), those from the urban site were mainly in gainful employment or self-employed—80.3% (Table 2).

**Table 2:** Characteristics of participants at enrolment into the CoLTART study by sex and study site

Characteristic	All combined	Entebbe site			Kyamulibwa site		
	n (%)	All, n (%)	Sex		All, n (%)	Sex	
			Females, n (%)	Males, n (%)		Females, n (%)	Males, n (%)
All by site	1095	666	459 (68.9)	207 (31.1)	429	252 (58.7)	177 (41.3)
Age, years							
18–34	127 (11.6)	34 (5.1)	29 (6.3)	5 (2.4)	93 (21.8)	65 (26.0)	28 (15.8)
35–49	649 (59.3)	426 (64.0)	295 (64.3)	131 (63.3)	222 (52.0)	126 (50.4)	96 (54.2)
50+	319 (29.1)	206 (30.9)	135 (29.4)	71 (34.3)	112 (26.2)	59 (23.6)	53 (30.0)
Mean age, years (SD)	44.8 (9.3)	46.1 (8.1)	45.5 (8.1)	47.3 (7.9)	42.7 (10.6)	41.6 (10.4)	44.3 (10.8)
Marital status							
Never married	87 (8.0)	28 (4.2)	24 (5.2)	4 (1.9)	59 (13.8)	40 (15.9)	19 (10.7)
Married/cohabiting	469 (42.8)	263 (39.5)	105 (22.9)	158 (76.3)	206 (48.0)	93 (36.9)	113 (63.8)
Separated/divorced	265 (24.2)	150 (22.5)	126 (27.5)	24 (11.6)	115 (26.8)	78 (31.0)	37 (20.9)
Widowed	274 (25.0)	225 (33.8)	204 (44.4)	21 (10.1)	49 (11.4)	41 (16.2)	8 (4.5)
Education level							
Incomplete primary	498 (45.5)	185 (27.8)	133 (29.0)	52 (25.1)	313 (73.0)	181 (71.8)	132 (74.6)
Complete primary	169 (15.4)	119 (17.9)	85 (18.5)	34 (16.4)	50 (11.6)	27 (10.7)	23 (13.0)
Secondary+	428 (39.1)	362 (54.3)	241 (52.5)	121 (58.5)	66 (15.4)	44 (17.5)	22 (12.4)
Employment^a^							
Peasant/farmer	362 (33.1)	64 (9.6)	45 (9.8)	19 (9.2)	298 (69.5)	179 (71.0)	119 (67.2)
Gainful employment	221 (20.2)	174 (26.2)	113 (24.7)	61 (29.5)	47 (11.0)	26 (10.3)	21 (11.9)
Self-employed/business	427 (39.0)	360 (54.1)	242 (52.8)	118 (57.0)	67 (15.6)	36 (14.3)	31 (17.5)
Unemployed	84 (7.7)	67 (10.1)	58 (12.7)	9 (4.3)	17 (3.9)	11 (4.4)	6 (3.4)
SES score tertile^b^							
Low	458 (42.0)	277 (41.9)	222 (48.9)	55 (26.6)	181 (42.2)	111 (44.0)	70 (39.5)
Middle	413 (37.9)	245 (37.1)	159 (35.0)	86 (41.5)	168 (39.2)	104 (41.3)	64 (36.2)
High	219 (20.1)	139 (21.0)	73 (16.1)	66 (31.9)	80 (18.6)	37 (14.7)	43 (24.3)
Tobacco consumption^c^							
Never	898 (82.1)	575 (86.4)	430 (93.8)	145 (70.0)	323 (75.2)	234 (92.8)	89 (50.3)
Ex-smoker	105 (9.6)	61 (9.2)	14 (3.1)	47 (22.7)	44 (10.3)	4 (1.6)	40 (22.6)
Current	91 (8.3)	29 (4.4)	14 (3.1)	15 (7.2)	62 (14.5)	14 (5.6)	48 (27.1)
Alcohol consumption^d^							
Never	408 (37.6)	248 (37.7)	186 (41.1)	62 (30.4)	160 (37.4)	112 (44.4)	48 (27.1)
Ever >1 month	383 (35.3)	255 (38.9)	183 (40.4)	72 (35.3)	128 (29.7)	80 (31.7)	48 (27.1)
Within <1 month	295 (27.1)	154 (23.4)	84 (18.5)	70 (34.3)	141 (32.9)	60 (23.9)	81 (45.8)
Work involves moderate/vigorous activity^e^							
No	494 (45.2)	433 (65.1)	333 (72.7)	100 (48.3)	61 (14.3)	48 (19.0)	13 (7.4)
Yes	599 (54.8)	232 (34.9)	125 (27.3)	107 (51.7)	367 (85.7)	204 (81.0)	163 (92.6)
Days/week-animal proteins^f^							
0	300 (27.6)	127 (19.3)	98 (21.6)	29 (14.1)	173 (40.3)	123 (48.8)	50 (28.2)
1 or 2	487 (44.8)	298 (45.3)	210 (46.4)	88 (42.9)	189 (44.1)	98 (38.9)	91 (51.4)
3+	300 (27.6)	233 (35.4)	145 (32.0)	88 (42.9)	67 (15.6)	31 (12.3)	36 (20.4)
Adds salt to food^g^							
No	625 (57.8)	399 (61.0)	283 (63.0)	116 (56.6)	226 (52.8)	148 (58.7)	78 (44.3)
Yes	457 (42.2)	255 (39.0)	166 (37.0)	89 (43.4)	202 (47.2)	104 (41.3)	98 (55.7)
Type of oil/fat used in cooking^h^							
None	146 (13.6)	68 (10.5)	43 (9.6)	25 (12.3)	78 (18.3)	54 (21.4)	24 (13.7)
Vegetable oil	793 (73.6)	488 (75.1)	334 (74.9)	154 (75.5)	305 (71.4)	183 (72.6)	122 (69.7)
Animal fat	111 (10.3)	73 (11.2)	53 (11.9)	20 (9.8)	38 (8.9)	13 (5.2)	25 (14.3)
Others/none in particular	27 (2.5)	21 (3.2)	16 (3.6)	5 (2.5)	6 (1.4)	2 (0.8)	4 (2.3)
Total teaspoons of sugar added to drink/day^i^							
<3	337 (31.5)	247 (38.4)	149 (33.7)	98 (48.8)	90 (21.0)	46 (18.3)	44 (25.0)
4/7	428 (39.9)	279 (43.4)	202 (45.7)	77 (38.3)	149 (34.8)	84 (33.3)	65 (36.9)
8+	306 (28.6)	117 (18.2)	91 (20.6)	26 (12.9)	189 (44.2)	122 (48.4)	67 (38.1)

*All combined* combines both Entebbe and Kyamulibwa sites. SES score tertile—Social Economic Status score computed from asset index based on household ownership of items

Number of participants with missing data: ^a^Employment—1, ^b^SES score tertile—5, ^c^Tobacco consumption—1, ^d^Alcohol consumption—9, ^e^Work involves moderate/vigorous activity—2, ^f^Days/week-animal proteins—8, ^g^Adds salt to food—13, ^h^Type of oil/fat used in cooking—18, ^i^Total teaspoons of sugar added to drink/day—24

Among our study population, the overall prevalence of hypertension was 14.5%, Diabetes mellitus—2.1% and renal disease—0.6%. Participants from the urban site had been on ART for a longer duration, with 90.7% on ART for 9 years and over compared to 11.7% at the rural site. Overall, 236 (23.1%) of our participants were on a PI containing ART regimen with a higher proportion of patients being at the urban Entebbe site; 32.4% compared to 7.6% among those at the rural Kyamulibwa site and 608 (59.6%) were or had ever been on a Tenofovir containing ART regimen; 78.3% of those at the urban site and 28.3% at the rural site (Table 3). We found that among individuals on long-term ART; (a) there were no differences in renal dysfunction (glomerular function and renal tubular function) between patients on Tenofovir containing and Non-Tenofovir containing ART regimens (Table 4). (b) Patients on PI containing ART regimens had higher total cholesterol, lower high density lipoprotein, higher low density lipoprotein, higher triglycerides, and a high atherogenic index for plasma than patients on non-PI containing ART regimen, p = 0.001 or < 0.001. (c) Patients on non-PI containing ART regimen had higher diastolic hypertension than patients on PI containing ART regimen, p < 0.001 (Table 5).

**Table 3:** Medical history of participants at enrolment into the CoLTART study by sex and site

Medical condition	All combined	Entebbe site			Kyamulibwa site		
	n (%)	All, n (%)	Sex		All, n (%)	Sex	
			Females, n (%)	Males, n (%)		Females, n (%)	Males, n (%)
All	1095	666	459 (68.9)	207 (31.1)	429	252 (58.7)	177 (41.3)
Hypertension history							
Never had hypertension	936 (85.5)	556 (83.5)	378 (82.4)	178 (86.0)	380 (88.6)	222 (88.1)	158 (89.3)
Ever had hypertension	159 (14.5)	110 (16.5)	81 (17.6)	29 (14.0)	49 (11.4)	30 (11.9)	19 (10.7)
Diabetes mellitus history^a^							
No known diabetes mellitus	1067 (97.9)	645 (97.3)	445 (97.4)	200 (97.1)	422 (98.8)	248 (98.8)	174 (98.9)
Known diabetes mellitus	23 (2.1)	18 (2.7)	12 (2.6)	6 (2.9)	5 (1.2)	3 (1.2)	2 (1.1)
Renal disease history^b^							
No renal disease	1087 (99.4)	659 (99.1)	456 (99.3)	203 (98.5)	428 (99.8)	252 (100.0)	176 (99.4)
Known renal disease	7 (0.6)	6 (0.9)	3 (0.7)	3 (1.5)	1 (0.2)	0 (0.0)	1 (0.6)
ART exposure, years							
ART naïve	74 (6.8)	27 (4.1)	21 (4.6)	6 (2.9)	47 (11.0)	22 (8.7)	25 (14.1)
<1 years	79 (7.2)	13 (2.0)	11 (2.4)	2 (1.0)	66 (15.4)	35 (13.9)	31 (17.5)
1 to <5 years	164 (15.0)	7 (1.1)	6 (1.3)	1 (0.5)	157 (36.6)	98 (38.9)	59 (33.3)
5 to <9 years	124 (11.3)	15 (2.3)	9 (2.0)	6 (2.9)	109 (25.4)	65 (25.8)	44 (24.9)
9+ years	654 (59.7)	604 (90.7)	412 (89.8)	192 (92.8)	50 (11.7)	32 (12.7)	18 (10.2)
Metabolic abnormalities study groups^c^							
Non-protease Inhibitor based ART	785 (76.9)	432 (67.6)	306 (69.9)	126 (62.7)	353 (92.4)	212 (92.2)	141 (92.8)
Protease Inhibitor based ART	236 (23.1)	207 (32.4)	132 (30.1)	75 (37.3)	29 (7.6)	18 (7.8)	11 (7.2)
Renal dysfunction study groups^d^							
Tenofovir containing ART	608 (59.6)	500 (78.3)	337 (76.9)	163 (81.1)	108 (28.3)	64 (27.8)	44 (29.0)
Non-Tenofovir containing ART	413 (40.5)	139 (21.8)	101 (23.1)	38 (18.9)	274 (71.7)	166 (72.2)	108 (71.1)
Body mass index (kg/m^2^)^e^							
<18.5	114 (10.6)	48 (7.4)	29 (6.5)	19 (9.4)	66 (15.4)	29 (11.5)	37 (21.0)
18.5–24.9	674 (62.8)	365 (56.6)	217 (49.0)	148 (73.3)	309 (72.2)	175 (69.4)	134 (76.1)
25.0–29.9	218 (20.3)	172 (26.7)	140 (31.6)	32 (15.8)	46 (10.8)	41 (16.3)	5 (2.8)
≥30	67 (6.3)	60 (9.3)	57 (12.9)	3 (1.5)	7 (1.6)	7 (2.8)	0 (0.0)
Haemoglobin (g/dl)^f^							
Normal	908 (83.3)	570 (86.2)	387 (85.1)	183 (88.8)	338 (78.8)	199 (79.0)	139 (78.5)
Abnormal	182 (16.7)	91 (13.8)	68 (14.9)	23 (11.2)	91 (21.2)	53 (21.0)	38 (21.5)
CD4 cell counts at enrolment (cells/µl)^g^							
≤350	307 (30.1)	182 (30.6)	102 (25.2)	80 (42.3)	125 (29.3)	65 (26.1)	60 (33.9)
351–500	327 (32.1)	171 (28.8)	118 (29.1)	53 (28.0)	156 (36.6)	89 (35.7)	67 (37.9)
501+	386 (37.8)	241 (40.6)	185 (45.7)	56 (29.6)	145 (34.0)	95 (38.2)	50 (28.2)
Viral loads at enrolment (cells/ml)^h^							
≤1000 copies/ml	823 (77.7)	528 (81.6)	363 (81.2)	165 (82.5)	295 (71.6)	180 (75.3)	115 (66.5)
>1000 copies/ml	236 (22.3)	119 (18.4)	84 (18.8)	35 (17.5)	117 (28.4)	59 (24.7)	58 (33.5)

Number of participants with missing data: ^a^Diabetes mellitus history—5, ^b^Renal disease history—1, ^c^Metabolic abnormalities study groups—74 patients who were ART naïve at enrolment, ^d^Renal dysfunction study groups—74 patients who were ART naïve at enrolment, ^e^Body Mass Index—22, ^f^Haemoglobin—5, ^g^CD4 cell counts at enrolment—75, ^h^Viral loads at enrolment-36; the 1059 included the 74 ART naïve individuals pending ART initiation

*NNRTI* nucleoside reverse transcriptase inhibitor, *NNRTI* non-nucleoside reverse transcriptase inhibitor

**Table 4:** Proportion of renal dysfunction/failure, and adjusted mean differences in renal function measures by TDF exposure among 964 adults on ART for more than 6 months

Renal function assessment parameter	Renal dysfunction/failure, n (%)	Adjusted mean differences (95% CI), non-TDF as reference	p value
eGFR (Cockcroft-Gault, adj for BSA)			
Non-TDF	99/378 (26.2%)	Ref	0.797
TDF	142/501 (28.3%)	−2.15 (−8.53 to 4.22)	
TDF-stopped	14/50 (28.0%)	−0.62 (−12.74 to 11.50)	
eGFR (Cockcroft-Gault, without BSA adj)			
Non-TDF	141/379 (37.2%)	Ref	0.914
TDF	196/502 (39.0%)	−2.42 (−14.45 to 9.62)	
TDF-stopped	19/50 (38.0%)	−3.01 (−25.90 to 19.89)	
eGFR (CKD-Epi)			
Non-TDF	60/383 (15.7%)	Ref	0.837
TDF	109/514 (21.2%)	−0.88 (−3.84 to 2.08)	
TDF-stopped	9/50 (18.0%)	−0.78 (−6.42 to 4.87)	
eGFR (MDRD with race)			
Non-TDF	28/383 (7.3%)	Ref	0.872
TDF	57/514 (11.1%)	−0.61 (−8.09 to 6.87)	
TDF-stopped	4/50 (8.0%)	3.11 (−11.14 to 17.36)	
Fractional tubular PO4 reabsorption			
Non-TDF	16/339 (4.7%)	Ref	0.131
TDF	16/386 (4.1%)	−0.74 (−1.84 to 0.36)	
TDF-stopped	1/42 (2.4%)	1.12 (−0.92 to 3.15)	
Serum urea			
Non-TDF	0/382 (0.0%)	Ref	0.252
TDF	2/514 (0.4%)	0.03 (−0.19 to 0.25)	
TDF-stopped	0/50 (0.0%)	0.35 (−0.07 to 0.77)	
Serum creatinine (μmol/l)			
Non-TDF	4/383 (1.0%)	Ref	0.336
TDF	10/514 (1.9%)	2.03 (−1.40 to 5.46)	
TDF-stopped	3/50 (6.0%)	3.98 (−2.55 to 10.51)	
Serum phosphates (mmol/l)			
Non-TDF	64/383 (16.7%)	Ref	0.575
TDF	50/503 (9.9%)	0.01 (−0.04 to 0.06)	
TDF-stopped	8/48 (16.7%)	−0.04 (−0.13 to 0.05)	

Mean difference adjusted for site, duration on ART, smoking status, social-economic status, log viral load, CD4 cell count, hypertension and Glucose; Renal dysfunction/failure defined as <90 for estimated Glomerular Filtration Rates, >11.9 mmol/l for serum Urea, >109 μmol/l for serum creatinine and <0.81 mmol/l for serum phosphates (Hypophosphataemia)

*eGFR* estimated glomerular filtration rate, *BSA* body surface area adjustment, *CKD-Epi* Chronic Kidney Disease Epidemiology formula, *MDRD* Modified Diet in Renal Disease formulae with race adjustment

**Table 5:** Proportion of abnormal values for CVD risk factors and differences in mean values of CVD risk measures by PI exposure among the 964 adults on ART for more than 6 months

CVD risk measure	Abnormal values for CVD risk, n (%)	ADJUSTED mean differences (95% CI), non-PI as reference	p value
Total cholesterol ≥5.2 μmol/l			
Non-PI regimen	176/714 (24.6%)	Ref	<0.001
PI regimen	121/230 (52.6%)	0.78 (0.57 to 0.98)	
HDL <1 mmol/l (males), <1.3 mmol/l (females)			
Non-PI regimen	265/714 (37.1%)	Ref	0.001
PI regimen	67/230 (29.1%)	0.12 (0.05 to 0.20)	
LDL ≥3.4 mmol/l			
Non-PI regimen	144/714 (18.6%)	Ref	<0.001
PI regimen	93/230 (40.3%)	0.45 (0.29 to 0.62)	
Total cholesterol/HDL ratio (>5.1)			
Non-PI regimen	90/714 (12.6%)	Ref	0.420
PI regimen	42/230 (18.3%)	0.10 (−0.15 to 0.35)	
Triglycerides ≥1.69 mmol/l			
Non-PI regimen	109/713 (15.3%)	Ref	<0.001
PI regimen	99/230 (43.0%)	0.52 (0.24 to 0.80)	
Glucose (>6.4 mmol/l)			
Non-PI regimen	26/714 (3.6%)	Ref	0.814
PI regimen	7/231 (3.0%)	0.00 (−0.03 to 0.04)	
Systolic blood pressure (≥140 mmHg)			
Non-PI regimen	110/707 (15.6%)	Ref	0.060
PI regimen	19/234 (8.1%)	−3.120 (−6.413 to 0.174)	
Diastolic blood pressure (≥90 mmHg)			
Non-PI regimen	112/707 (15.8%)	Ref	<0.001
PI regimen	13/234 (5.6%)	−3.931 (−5.967 to 1.895)	
AIP log_10_ (Triglycerides/HDL) ≥0.1			
Non-PI regimen	183/713 (25.7%)	Ref	<0.001
PI regimen	102/230 (44.3%)	0.12 (0.07 to 0.17)	
Abnormal BMI (≥30 kg/m^2^)			
Non-PI regimen	44/703 (6.3%)	Ref	0.074
PI regimen	17/231 (7.3%)	−2.370 (−5.005 to 0.264)	
Abnormal waist circumference [≥94 cm (men)/≥80 (women)]			
Non-PI regimen	287/706 (40.6%)	Ref	0.805
PI regimen	94/234 (40.2%)	−0.202 (−1.829 to 1.425)	
Abnormal waist/hips ratio [>0.95 (men)/>85 (women)]			
Non-PI regimen	369/706 (52.3%)	Ref	0.118
PI regimen	133/234 (56.8%)	0.008 (−0.002 to 0.019)	
Abnormal Framingham score (>10%)			
Non-PI regimen	130/703 (18.5%)	Ref	0.434
PI regimen	28/229 (12.2%)	−0.344 (−1.218 to 0.530)	

Mean differences adjusted for site, duration on ART, sex, age-groups, smoking status, alcohol consumption, days/week-animal proteins, salt and sugar intake, physical activity, social-economic status, log viral load, CD4 cell count

*HDL* high density lipoprotein, *LDL Low Density Lipoprotein, AIP* atherogenic index for plasma, *BMI* body mass index

## Discussion

In sub Saharan Africa, there is paucity of data on the complications of long-term ART use generated from the region. The CoLTART cohort therefore provides a good platform to investigate the complications of long-term ART in a Ugandan population. In this cross-sectional analysis of data collected at enrolment of patients who had been on ART for a median of 9.4 years, we found no differences in renal function (eGFR and fractional tubular phosphate reabsorption) between patients on Tenofovir and Non-Tenofovir containing ART regimens. However, we found that patients on PI containing ART regimens had higher cardiometabolic risk factors including higher total cholesterol, lower high density lipoprotein, higher low density lipoprotein, higher triglycerides, and a high atherogenic index for plasma compared to the non-PI regimen.

These findings are reassuring against concerns about Tenofovir induced renal toxicity, especially as it is now recommended by WHO for initiating ART, and has been widely adopted by ART programmes in many resource limited countries including Uganda [12–15]. Short to medium follow up studies have reported an association between Tenofovir and renal dysfunction, leading to glomerular and proximal renal tubular damage and acute renal failure [17–20]. Our failure to find differences in renal dysfunction by Tenofovir use might be due to the fact that most of our patients had been on ART for more than 9 years. Similarly, other previous studies have reported that the loss in eGFR attributable to Tenofovir seemed to occur during the early years of exposure between 0.5 and 31.2 months and stabilized after that [38–41].

As HIV infected individuals live longer due to improved survival on ART, cardiometabolic co-morbidity will manifest, and the association of HIV infection and cardiovascular disease will be compounded by ART use. The association of PI containing ART regimen and cardiometabolic risk is important since PIs are the backbone of second line ART regimens in resource limited settings. With the advocacy and availability of virological monitoring of patients on ART, more patients with virological failure will be identified and switched to PI containing second line ART regimens. HIV care programmes in resource limited settings therefore need to integrate CVD risk assessment and preventive measures, including routine biochemical and biophysical monitoring as well as stocking of lipid lowering drugs, increasing physical exercises, dietary adjustments.

### Study strengths

The CoLTART cohort study participants have been on ART for close to a decade and thus enables assessment of the temporal relationship between ART and ART complications. The DART and RCC cohorts have systematically collected data on clinical, immunological and virological treatment outcomes, ARV toxicities, with regular haematology, liver and kidney function biochemical test results that can be used retrospectively. Stored serum and plasma samples that can be used for various analyses for the status during the early ART period (DART) and during the Pre-ART and early ART periods (RCC) are also available. The MRC/UVRI Uganda Unit’s CDLS has the capacity for analysing and storing research samples.

### Study weakness

Study participants of the former cohorts may not be representative of the general HIV-positive population, and those who were available for enrolment may be a biased sample due to their longer survival. The study included limited outcomes, other long-term ART complications, such as reduced bone mineral density and osteoporosis were not studied due to the costs of the non-invasive DXA (DECK-sa) scan, a procedure used to measure bone density, as well as mineral content in the body. In our study, a small number of patients; 50 (8.9%) had discontinued Tenofovir but we did not consider the period since Tenofovir was discontinued and the reasons why it was discontinued. If Tenofovir was stopped due to Tenofovir induced renal dysfunction, this might have biased our results. Due to the low prevalence of antibody seropositivity and confirmed Hepatitis C virus (HCV) RNA infection in Uganda [42, 43], we did not screen for HCV in this study. Other potential sources of bias included the self-reported thus subjectively measured individual level exposures especially dietary, socio-economic and behavioural factors. There might have been some inaccuracies in tracking time on particular regimens of ART e.g. the exact dates of ART switches or substitutions.

## Conclusions

Our findings allay fears of Tenofovir renal toxicity and the WHO recommendation to use Tenofovir in first line ART can safely be implemented even in resource limited settings with limited renal function monitoring. However, further evaluation of renal function among patients on Tenofovir beyond 10 years is advised. Integration of cardiovascular risk assessment, institution of preventive and curative or control remedies against cardiovascular disease are recommended.

## Data available

Data available includes demographic, social economic status, medical, dietary and lifestyle risk factors. Biophysical measurements data available includes weight, height, blood pressure and pulse rate; waist, hip and mid-upper arm circumferences. Clinical examination and ART and cotrimoxazole/dapsone adherence data is also available. Available laboratory data includes: fasting blood glucose and lipid profiles (total cholesterol, triglycerides, low density lipoproteins, high density lipoproteins); renal function tests (Urine strip test, phosphates and creatinine and serum phosphates, creatinine and urea). Full blood counts, immunology (CD3, CD4 and CD8), viral loads and HIV drug resistance test results for blood samples with viral loads ≥1000 copies/ml. Liver function tests (alanine transaminase, aspartate transaminase, alkaline phosphatase and bilirubin). Stored samples are also available for Hepatitis B virus serology, genomic studies and other studies.

## Data resource access

The CoLTART database has data on the participants biophysical measurements, clinical examination findings and laboratory measurements. All data from the cohort are managed by the Statistics Section of the MRC/UVRI Uganda Research Unit on AIDS. Data access inquiries can be made to the Director, MRC/UVRI, Uganda Research Unit on AIDS by email to: mrc@mrcuganda.org or the corresponding author.

## Abbreviations

AIDS: Acquired Immune Deficiency Syndrome
ART: antiretroviral therapy
ARVs: antiretroviral drugs
CAP: College of American Pathologists
CDLS: Clinical Diagnostics Laboratory Services
CRFs: case report forms
CoLTART: Complications of Long-Term Antiretroviral Therapy
CPR: calibrated population resistance tool
CVD: cardiovascular disease
DART: Development of Antiretroviral therapy in Africa
DXA: dual-energy X-ray absorptiometry
EDTA: ethylenediaminetetraacetic acid
eGFR: estimated glomerular filtration rate
GCP: Good Clinical Practice
HIV: human immunodeficiency virus
ICH: International Council for Harmonisation
MRC: Medical Research Council
NRTI: Nucleoside Reverse Transcriptase Inhibitors
NNRTI: Non Nucleoside Reverse Transcriptase Inhibitors
PCR: Polymerase Chain Reaction
PI: protease inhibitor
RCC: Rural Clinical Cohort
RCPA: Royal College of Pathologists of Australasia
RMD: Roche Molecular Diagnostics
RNA: ribonucleic acid
SDRMs: surveillance drug resistance mutations
STEPS: STEPwise approach to surveillance
TDF: Tenofovir Disoproxil Fumarate
TDR: transmitted drug resistance
UKNEQAS: United Kingdom National External Quality Assurance Service
UVRI: Uganda Virus Research Institute
WHO: World Health Organisation
